# Effect of planned visual education on university students’ attitudes and beliefs regarding skin cancer: a cluster-randomized controlled trial

**DOI:** 10.1590/1516-3180.2024.0252.R1.14022025

**Published:** 2025-05-30

**Authors:** Esin Sevgi Dogan, Ozden Dedeli Caydam

**Affiliations:** I Department of Nursing, Manisa Celal Bayar University, Manisa, Türkiye.; II Department of Nursing, Manisa Celal Bayar University, Manisa, Türkiye.

**Keywords:** Skin neoplasms, Health belief model, Students, Health attitudes, Health beliefs, University student, Skin cancer

## Abstract

**BACKGROUND:**

The incidence of skin cancer is increasing globally. However, it is largely preventable through early detection. Therefore, raising public awareness through education is essential.

**OBJECTIVE:**

This study aimed to evaluate the effect of a planned visual education program—based on the Health Belief Model—on university students’ attitudes and beliefs regarding skin cancer.

**DESIGN AND SETTING:**

This cluster-randomized controlled trial was conducted in two departments at a university in Manisa, Türkiye.

**METHODS:**

The study was conducted with 116 university students, divided equally into an intervention group (n = 58) and a control group (n = 58). Data were collected using the Student Information Form and the Health Belief Model Scale in Skin Cancer.

**RESULTS:**

Following the visual education program based on the Health Belief Model, significant differences were observed between the intervention and control groups in perceived severity, perceived susceptibility, perceived barriers, perceived benefits, and self-efficacy scores. While the intervention group showed significant improvements across these domains, no significant difference was found in perceived severity scores.

**CONCLUSION:**

The findings indicate that planned visual education based on the Health Belief Model positively influenced students’ attitudes and beliefs regarding skin cancer.

**CLINICAL TRIAL REGISTRATION:**

The research was recorded at https://clinicaltrials.gov/study/NCT05788939.

## INTRODUCTION

The incidence rate of skin cancer is steadily increasing both worldwide and in Türkiye.^1,2^ According to 2014 data, the incidence was 28.3 per 100,000 in men and 18 per 100,000 in women.^3^ Skin cancer is the most common type of cancer among individuals aged 25–29 years and the second most common type in those aged 15–29 years.^4,5^ Melanoma—the most fatal form of skin cancer—is particularly prevalent among adolescents and young adults.^6^ Therefore, primary prevention strategies for skin cancer should specifically target this population.^7,8^

The most significant risk factor for skin cancer is exposure to ultraviolet (UV) radiation.^9,10^ Youth and childhood are the most vulnerable periods for skin damage and UV exposure. The amount of UV radiation absorbed during these years substantially contributes to skin cancer risk in adulthood. Additionally, approximately 25–50% of an individual’s lifetime sun and UV exposure occurs between the ages of 18 and 21.^10,11^ Young people frequently engage in outdoor social activities that further increase their exposure to sunlight.^12^

Adolescence is a developmental stage marked by substantial physical, psychological, and social change during which lifelong health behaviors are established.^13^ Young individuals may struggle to adapt to these changes. Although skin cancer is highly preventable through proper sun protection measures, studies indicate that young people often do not adhere to recommended protective behaviors.^9,10,11^ Consequently, it is essential to implement educational initiatives aimed at skin cancer prevention among young people.^14^

To promote healthy behaviors and improve sun protection and skin cancer prevention practices among young individuals, visual education campaigns incorporating videos, brochures, and posters that reinforce learning are recommended.^15^ In European countries, such campaigns are implemented to raise awareness and knowledge about skin cancer and the risks associated with sun exposure.^16,17,18^ However, few studies have focused on university students,^18,19,20,21,22^ and limited research has explored the use of visual education programs incorporating digital applications to increase skin cancer awareness in Türkiye.^23,24^

Developing preventive behaviors for skin cancer requires individuals to reflect on their attitudes and beliefs regarding the disease.^5,9^ The Health Belief Model (HBM)—a widely used framework in health promotion—proposes that health behaviors are influenced by individuals’ beliefs and perceptions. Although the HBM is frequently applied to explain and guide preventive health behaviors,^25^ the number of studies investigating its use in the context of skin cancer^26,27,28,29^ is limited and largely descriptive.^30^ To our knowledge, no studies have examined the impact of a planned visual education program based on the HBM on university students’ attitudes and beliefs about skin cancer. Therefore, this study was conducted to assess the effects of such an intervention.

## OBJECTIVE

The aim of this study was to assess the effects of a planned visual education program—based on the Health Belief Model—on university students’ attitudes and beliefs about skin cancer.

## METHODS

This study was conducted as a cluster-randomized controlled trial. The sample comprised 116 second-year students at Manisa Celal Bayar University (MCBU). Participants were enrolled in the Faculty of Economics and Administrative Sciences and the Faculty of Technology. The study was conducted between February and September 2019. Simple random sampling was used to determine the sample. A draw was conducted among the faculties at MCBU. The Faculty of Economics and Administrative Sciences was assigned as the intervention group (IG), and the Faculty of Technology was assigned as the control group (CG). Among the seven departments within the Faculty of Economics and Administrative Sciences and the four departments within the Faculty of Technology, lots were drawn again using simple random sampling. The Department of Econometrics was assigned as the IG, and the Department of Mechatronics Engineering was assigned as the CG. The research was conducted as a single-blind study in which participants were blinded; they did not know whether they belonged to the IG or CG.

A power analysis was conducted to determine an adequate sample size that would yield reliable results and allow for statistical analysis. Based on repeated-measures analysis of variance using the G*Power 3.1 software (Heinrich Heine University, Düsseldorf), the minimum required sample size was calculated to be 51 participants per group, assuming 80% power, a significance level of 0.05, and a medium effect size (0.5) with two repeated measurements. A total of 58 students were recruited for each group. The post hoc power of the study was computed as 0.95 at a significance level of α = 0.05.

The inclusion criteria were as follows: being 18 years of age or older, actively enrolled in formal education and attending classes, and voluntarily agreeing to participate in the study.

### Ethical standards

Approval was obtained from the Ethics Committee of the Faculty of Medicine, Manisa Celal Bayar University Health Sciences (dated 27/06/2018; protocol no. 20.478.486), along with institutional permissions, prior to conducting the study. This research was carried out in accordance with the principles of the Declaration of Helsinki. The aim and procedure of the study were explained to the students, and informed consent forms were signed. Upon completion of the study, the CG received the same educational content, including slides and videos, and project-related keychains were distributed.

## HYPOTHESES

- Hypothesis 1 (H1-1): There is a difference between the IG and CG in perceived susceptibility to skin cancer as a significant health issue.
- Hypothesis 2 (H1-2): There is a difference between the IG and CG in their beliefs about developing skin cancer and its consequences.
- Hypothesis 3 (H1-3): There is a difference between the IG and CG in their beliefs that recommendations for preventing skin cancer are useful.
- Hypothesis 4 (H1-4): There is a difference between the IG and CG in perceived barriers to preventing skin cancer.
- Hypothesis 5 (H1-5): There is a difference between the IG and CG in their confidence in taking preventive measures against skin cancer.
- Hypothesis 6 (H1-6): There is a time-based difference within the IG in perceived susceptibility to skin cancer.
- Hypothesis 7 (H1-7): There is a time-based difference within the IG in beliefs about developing skin cancer and its consequences.
- Hypothesis 8 (H1-8): There is a time-based difference within the IG in beliefs that recommendations for preventing skin cancer are useful.
- Hypothesis 9 (H1-9): There is a time-based difference within the IG in perceived barriers to preventing skin cancer.
- Hypothesis 10 (H1-10): There is a time-based difference within the IG in confidence regarding taking preventive measures against skin cancer.

### Instruments

The study data were collected using the Student Information Form and the HBM Scale in Skin Cancer.

### The Student Information Form

This form was developed by the researchers based on a review of the relevant literature.^20,21,31^ It consists of 24 questions concerning participants’ sociodemographic characteristics.

### The Health Belief Model Scale in Skin Cancer (HBMSSC)

The scale was developed by Dogan and Caydam.^32^ The HBMSSC includes five sub-dimensions: perceived benefit, perceived susceptibility, perceived barriers, perceived severity, and self-efficacy. It comprises 26 items and is a Likert-type scale. Each item is rated on a scale from 1 (“strongly disagree”) to 5 (“strongly agree”). The total Cronbach’s alpha coefficient for the scale is 0.87. The Cronbach’s alpha coefficients for the five sub-dimensions are 0.79, 0.89, 0.65, 0.77, and 0.86, respectively. The “perceived barriers” sub-dimension is reverse-coded. The HBMSSC does not calculate a total score; instead, scores are derived independently for each sub-dimension. Higher scores in the “perceived severity,” “perceived benefit,” “perceived susceptibility,” and “self-efficacy” sub-dimensions indicate stronger perceptions in each respective domain.^32^ In this study, the Cronbach’s alpha coefficient for the total HBMSSC was 0.89 in the intervention group. The sub-dimension coefficients were 0.86, 0.94, 0.81, 0.71, and 0.92, respectively. In the control group, the total Cronbach’s alpha was 0.78, and the sub-dimension values were 0.86, 0.81, 0.89, 0.75, and 0.90, respectively.

### Data collection

**Figure 1:**
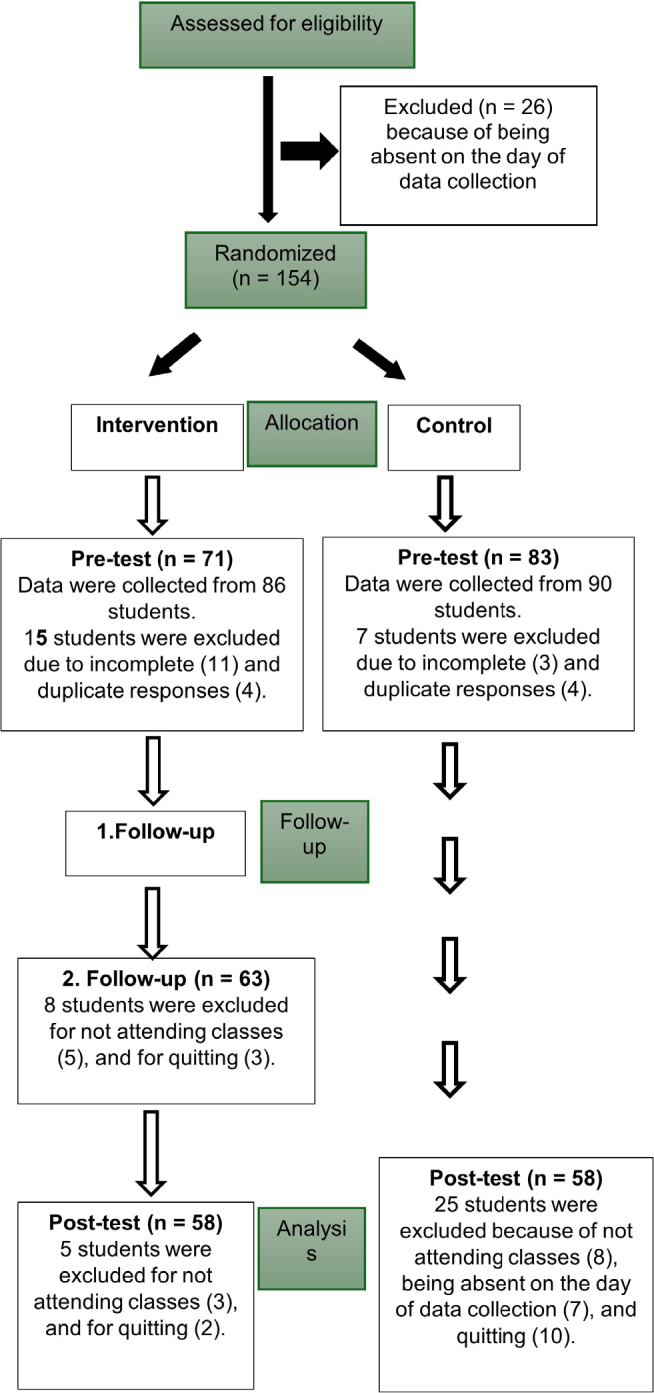
Flow diagram of the study.

Data were collected twice from the CG: before the training (pre-test) and seven months after the training (post-test). For the IG, data were collected four times: before the planned visual education program (pre-test), at the first and third months during the follow-up period, and at seven months post-intervention (post-test) (Figure 1).

### Planned visual education program

The educational materials included a PowerPoint presentation, two videos titled Mr. Sun and Dear 16*-* Year-Old Me, a skin cancer model, three posters, and a brochure. Professional support was obtained from a media company to produce subtitled versions of the Mr. Sun video. The researcher presented the content of the presentation slides, brochure, and posters.

Pre-test: The forms were distributed to students, who were asked to complete them. The presentation and skin cancer model were shown, and brochures were handed out.

1st Month: Students completed the HBMSSC. The videos were shown, and bandanas—intended to be worn as bracelets—were distributed. An Instagram page (@derikanseri), created for the campaign, was introduced. Students were informed that the first 20 people who followed the page, took a photo wearing the bandana, and tagged the page would receive gift items (sunscreen for the first 10 participants and beach towels for the next 10). Posters were displayed throughout the faculty building. A WhatsApp group was created to send periodic reminder messages to participants.

3rd Month: Students again completed the HBMSSC. Participants who followed the Instagram page received their designated gifts. Students were reminded to stay engaged during the summer and were informed that new surprises would be announced in September. Skin cancer awareness messages were sent monthly via Instagram, WhatsApp, and text message applications in June, July, and August.

Post-test: Students completed the final HBMSSC. They were informed of the study’s conclusion, and all participants received a gift—a keychain designed by the researchers featuring sun protection imagery.

### Statistical analysis

SPSS version 21.0 (IBM Corp., Armonk, NY, USA) was used for data analysis. Descriptive statistics were presented as mean ± standard deviation and frequency. To determine the suitability of the data for normal distribution, the decision was based on the “mean ± 2 standard deviations” rule, where 95.44% of the data were considered to fall within this range, indicating a normal distribution. As the data met the assumption of normality, parametric tests were applied. Between-group differences were analyzed using the independent samples t-test, while within-group differences were evaluated using the paired samples t-test. Repeated-measures analysis of variance was used when comparing more than two time points. Categorical variables were assessed using the chi-square test. Statistical significance was set at p < 0.05, with a 5% margin of error. A priori power analysis was conducted using G*Power version 3.1 (Heinrich Heine University, Düsseldorf) to determine the minimum required sample size.^33^ Statistical consultation was obtained from a professional data analysis company.

## RESULTS

No significant differences were observed between the groups in terms of sociodemographic characteristics or skin type (p > 0.05) (Table 1).

**Table 1:** Sociodemographic characteristics and skin type profiles of students in the intervention and control groups (n = 116)

	Intervention Group (n = 58)		Control Group (n = 58)		Significance
	$\bar{\text{x}}$ ± SD	Min-Max	$\bar{\text{x}}$ ± SD	Min-Max	
**Age (years)**	20.84 ± 1.08	19-24	20.91 ± 1.18	19-24	t = -0.326 p = 0.745
**Gender**	**n**	**%**	**n**	**%**	
*Female*	35	60.3	28	51.7	x^2^ = 1.702 p = 0.192
*Male*	23	39.7	30	48.3	
**Place of long-term residence**					
*Village/province*	22	37.9	30	51.7	x^2^ = 2.231 p = 0.135
*City*	36	62.1	28	47.3	
**Sunbelt of residence**					
*First belt*	47	81.1	39	67.2	x^2^ = 2.878 p = 0.090
*Second, third, and fourth belts*	11	18.9	19	32.8	
**Paternal education level**					
*Primary school*	14	24.1	15	25.9	x^2^ = 1.761 p = 0.623
*Secondary school*	15	25.9	11	18.9	
*High school*	19	32.8	17	29.3	
*University*	10	17.2	15	25.9	
**Paternal occupation**					
*Civil servant*	11	19.0	22	37.9	x^2^ = 6.701 p = 0.082
*Retired*	12	20.7	7	12.1	
*Self-employed*	18	31.0	11	19.0	
*Worker*	17	29.3	18	31.0	
**Maternal education level**					
*Primary school*	22	37.9	20	34.5	x^2^ = 0.459 p = 0.928
*Secondary school*	15	25.9	15	25.9	
*High school*	15	25.9	18	31.0	
*University*	6	10.3	5	8.6	
**Maternal occupation**					
*Civil servant*	5	8.6	5	8.6	x^2^ = 0.136 p = 0.987
*Retired*	6	10.4	7	12.1	
*Worker*	9	15.5	8	13.8	
*Housewife*	38	65.5	38	65.5	
**Perceived income**					
*Income* ≤ *expenses*	46	79.3	47	81.0	x^2^ = 0.054 p = 0.816
*Income > expenses*	12	20.7	11	19.0	
**Hair color**					
*Fair*	5	8.6	7	12.1	x^2^ = 0.402 p = 0.818
*Brown*	30	51.7	28	48.3	
*Black*	23	39.7	23	39.6	
**Eye color**					
*Blue/Green/Hazel*	15	25.9	13	22.5	x^2^ = 0.195 p = 0.907
*Brown*	37	63.8	39	67.2	
*Black*	6	10.3	6	10.3	
**Skin color**					
*Fair*	20	34.5	19	32.7	x^2^ = 0.143 p = 0.931
*Auburn/Light brown*	26	44.8	28	48.3	
*Brown/Brunette*	12	20.7	11	19.0	
**Skin type**					
*Burns easily, does not tan or tans very little*	21	36.2	12	20.7	x^2^ = 5.417 p = 0.114
*Burns, tans over time*	12	20.7	20	34.5	
*Burns very little, tans quickly*	12	20.7	16	27.6	
*Does not burn, tans quickly*	13	22.4	10	17.2	
**Birthmark status on the skin**					
*Yes*	22	37.9	18	31.0	x^2^ = 0.611 p = 0.415
*No*	36	62.1	40	69.0	
**History of sunburn in the last 12 months**					
*No*	23	39.7	26	44.9	x^2^ = 0.323 p = 0.851
*Once*	20	34.5	18	31.0	
*Twice or more*	15	25.8	14	24.1	
	** $\bar{\text{x}}$ ± SD**	**Min-Max**	** $\bar{\text{x}}$ ± SD**	**Min-Max**	
**Time in the sun between 10:00 a.m. and 4:00 p.m. in summer (hours)**	2.51 ± 1.21	0.5-6	2.78 ± 1.62	0-6	t = -1.004 p = 0.371
**Number of nevus on the skin**	16.53 ± 22.61	0-100	19.73 ± 24.63	0-150	t = -0.742 p = 0.46

Max = maximum; Min = minimum; SD = standard deviation; t = independent samples t-test; $\bar{\text{x}}$ = Mean; x^2^ = chi-square test; a p < 0.05.

While there were no significant differences in pre-test scores between the groups for perceived severity, perceived barriers, perceived susceptibility, perceived benefit, and self-efficacy (p > 0.05), significant differences were found in post-test scores (p < 0.01; p < 0.001; p < 0.001; p < 0.0001; and p < 0.0001, respectively). No significant differences were observed between the pre-test and post-test scores for any of the five dimensions in the control group (p > 0.05) (Table 2).

**Table 2:** Comparison of mean scores on the health belief model scale in skin cancer between intervention and control groups (n = 116)

Sub-dimension	Group	Pre-test (1st Month)		Post-test (7th Month)		Between-Group Significance	
		$\bar{\text{x}}$ ± SD		$\bar{\text{x}}$ ± SD		t	p
**Perceived Susceptibility**	Intervention	24.86 ± 6.02		27.01 ± 2.76		**-2.567**	**0.013** ^a^
	Control	24.94 ± 5.18		24.05 ± 5.52		0.901	0.370
	**Significance**	t	P	**t**	**P**		
		0.083	0.934	**3.654**	**0.000** ^c^		
**Perceived Benefit**	Intervention	20.56 ± 5.49		24.81 ± 3.27		**-5.364**	**0.000** ^c^
	Control	20.50 ± 4.40		20.53 ± 4.04		-0.044	0.965
	**Significance**	t	P	**t**	**P**		
		0.075	0.941	**6.106**	**0.000** ^c^		
**Perceived Severity**	Intervention	16.22 ± 3.19		17.12 ± 2.36		-1.914	0.061
	Control	15.18 ± 3.77		15.39 ± 3.95		-0.288	0.774
	**Significance**	t	P	**t**	**P**		
		1.544	0.114	**2.850**	**0.005** ^b^		
**Perceived Barriers**	Intervention	13.67 ± 3.93		16.15 ± 2.61		**-4.335**	**0.000** ^c^
	Control	14.48 ± 3.50		13.67 ± 3.15		1.849	0.070
	**Significance**	t	P	**t**	**P**		
		-1.239	0.218	**4.616**	**0.000** ^c^		
**Self-efficacy**	Intervention	23.53 ± 5.47		25.15 ± 3.51		**-2.063**	**0.044** ^a^
	Control	22.72 ± 5.35		21.84 ± 4.84		1.440	0.155
	**Significance**	t	P	**t**	**P**		
		0.980	0.422	**0.211**	**0.000** ^c^		

SD = standard deviation; t = independent samples t-test; $\bar{\text{x}}$ = Mean;

^a^ p < 0.05;

^b^ p < 0.01;

^c^ p < 0.001

Although the difference in perceived severity scores between the pre-test and post-test in the IG was not statistically significant (p > 0.05), the mean post-test score was higher than the pre-test score. Significant differences were observed between pre-test and post-test mean scores in the IG for perceived benefit (p < 0.001), perceived susceptibility (p < 0.05), perceived barriers (p < 0.05), and self-efficacy (p < 0.001) (Table 2).

Significant differences were found across follow-up measurements in the IG for perceived barriers (p < 0.01), perceived susceptibility (p < 0.05), perceived benefit (p < 0.001), and self-efficacy (p < 0.05). However, no significant difference was observed in perceived severity scores across follow-ups (p > 0.05) (Table 3).

**Table 3:** Within-group comparison of mean scores on the Health Belief Model Scale in Skin Cancer at pre-test, 1st, 3rd, and 7th months in the intervention group (n = 58)

Sub-dimension	Follow ups	$\bar{\text{x}}$ ± SD	Pairwise Comparisons					
			I		t	p	F	p
**Perceived Susceptibility**	Pre-test (I1)	24.86 ± 6.02	I 1-2	I1 < I2	-1.561	0.124	**3.298**	**0.027** ^a^
	1st month (I2)	26.20 ± 2.97	I 1-3	I1 < I3	**-2.292**	**0.026**		
	3rd month (I3)	26.91 ± 2.62	I 1-4	I1 < I4	**-2.567**	**0.013**		
	7th month (I4)	27.01 ± 2.76	I 2-3	I2 < I3	**-2.362**	**0.022**	**E.S.**	
			I 2-4	I2 < I4	-1.281	0.205	0.152	
			I 3-4	I3 < I4	-0.171	0.864		
**Perceived Benefit**	Pre-test (I1)	20.56 ± 5.49	I 1-2	I1 < I2	**-3.576**	**0.001**	**12.345**	**0.000** ^c^
	1st month (I2)	23.68 ± 4.36	I 1-3	I1 < I3	**-7.686**	**0.000**		
	3rd month (I3)	24.89 ± 3.30	I 1-4	I1 < I4	**-5.364**	**0.000**		
	7th month (I4)	24.81 ± 3.27	I 2-3	I2 < I3	**-2.820**	**0.007**	**E.S.**	
			I 2-4	I2 < I4	-1.480	0.144	0.402	
			I 3-4	I3 > I4	0.136	0.892		
**Perceived Severity**	Pre-test (I1)	16.22 ± 3.19	I 1-2	I1 < I2	-0.422	0.674	2.724	0.053
	1st month (I2)	16.41 ± 2.69	I 1-3	I1 < I3	-1.955	0.056		
	3rd month (I3)	17.08 ± 2.52	I 1-4	I1 < I4	-1.914	0.061		
	7th month (I4)	17.12 ± 2ç36	I 2-3	I2 < I3	**-2.413**	**0.019**	**E.S.**	
			I 2-4	I2 < I4	-1.769	0.082	0.129	
			I 3-4	I3 < I4	-0.100	0.920		
**Perceived Barriers**	Pre-test (I1)	13.67 ± 3.93	I 1-2	I1 < I2	-1.852	0.069	**6.637**	**0.001** ^b^
	1st month (I2)	14.82 ± 2.90	I 1-3	I1 < I3	**-3.178**	**0.002**		
	3rd month (I3)	15.62 ± 2.28	I 1-4	I1 < I4	**-4.335**	**0.000**		
	7th month (I4)	16.15 ± 2.61	I 2-3	I2 < I3	**-2.165**	**0.035**	**E.S.**	
			I 2-4	I2 < I4	**-2.463**	**0.017**	0.266	
			I 3-4	I3 < I4	-1.209	0.232		
**Self-efficacy**	Pre-test (I1)	23.53 ± 5.47	I 1-2	I1 < I2	-0.468	0.642	3.420	**0.023** ^a^
	1st month (I2)	23.91 ± 3.38	I 1-3	I1 < I3	-0.463	0.149		
	3rd month (I3)	24.67 ± 3.23	I 1-4	I1 < I4	**-2.063**	**0.044**		
	7th month (I4)	25.15 ± 3.51	I 2-3	I2 < I3	**-2.297**	**0.025**	**E.S.**	
			I 2-4	I2 < I4	**-2.233**	**0.023**	0.157	
			I 3-4	I3 < I4	-0.969	0.337		

E.S. = effect size; F = Pillai’s Trace test; I = intervention; SD = standard deviation; t = independent samples t-test; $\bar{\text{x}}$ = Mean;

^a^ p < 0.05;

^b^ p < 0.01;

^c^ p < 0.001.

## DISCUSSION

*Perceived susceptibility* refers to the extent to which individuals believe they are at risk of a disease or condition, with higher perceived risk typically associated with healthier behaviors.^25,34^ A significant difference was found in perceived susceptibility scores between the groups in the post-test (confirming H1-1) and across follow-ups within the IG (confirming H6-1). Similarly, studies on skin cancer in farmers^31^ and osteoporosis in women reported^35^ differences in perceived susceptibility during follow-ups after training. In contrast to our findings, a previous study^36^ on breast self-examination among university students found no significant difference in perceived susceptibility scores during follow-up. This discrepancy may be explained by the fact that the sample in the referenced study consisted of nursing students.

A significant difference in perceived susceptibility scores was also observed between the first and third months. In this context, incorporating video content into educational programs may help promote behavioral changes to prevent skin cancer. Existing literature supports this approach.^15,37,38^

*Perceived benefit* is defined as an individual’s belief that a particular behavior will effectively prevent a disease.^25,34^ A significant difference was observed in perceived benefit scores between groups in the post-test (confirming H3-1) and across follow-ups within the IG (confirming H8-1). Similar to our findings, one study reported differences in perceived benefit scores among farmers across follow-ups.^28^ In our study, the difference in scores appeared primarily between the first and third months, following delivery of the intervention via slides, brochures, and videos. These results suggest that visual education based on the HBM strengthened students’ beliefs in the usefulness of recommendations for skin cancer prevention. Supporting evidence in the literature indicates that visual education increases young people’s knowledge and awareness of skin cancer,^15^ improves sun protection and prevention behaviors,^15^ and enhances skin self-examination practices.^21^

*Perceived severity* refers to the extent to which an individual considers the consequences of a disease to be serious, with stronger perceptions generally associated with increased health-protective behaviors.^25,34^ A significant difference was observed in post-test perceived severity scores between the groups in this study (confirming H2-1). However, no significant change was found in perceived severity scores within the IG across time (rejecting H7-1). In contrast to these findings, a previous study reported significant changes in farmers’ perceived severity scores during follow-up,^29^ which may be attributed to the younger age of participants in our sample. Another study also found that visual education based on the HBM did not significantly affect perceived severity scores related to breast cancer prevention among university students.^36^

*Perceived barriers* refer to an individual’s beliefs about obstacles that may prevent them from adopting health behaviors.^25,34^ A significant difference was found in post-test perceived barriers scores between groups (confirming H4-1) and across follow-ups within the IG (confirming H9-1). Our findings are consistent with previous studies.^29,35^ These results suggest that HBM-based training reduced perceived barriers to skin cancer prevention. Notably, perceived barrier scores decreased following the video-based component of the training. Armstrong et al.^15^ found that video-based education was a more effective teaching tool than written materials for promoting sun protection among young people. Therefore, incorporating video-based educational materials into behavior change programs for skin cancer prevention appears to be beneficial.

*Self-efficacy* refers to an individual’s confidence in their ability to take action.^25,34^ A significant difference was observed in post-test self-efficacy scores between the groups (confirming H5-1) and across follow-ups within the IG (confirming H10-1). These findings are consistent with previous studies on skin cancer in farmers^29^ and osteoporosis in women.^35^ In another study involving workers, a positive change in knowledge and attitudes toward skin cancer was observed following a planned visual education program.^39^ Accordingly, it can be concluded that planned visual training increased individuals’ confidence and knowledge regarding the ability to engage in preventive behaviors for skin cancer.

The difference in mean self-efficacy scores across follow-up periods appears to be associated with the activities implemented between the pre-test and post-test. Specifically, the improvement was observed following the delivery of video-based training to the IG. Prior research has shown that video-based education is more effective than written materials and enhances both sunscreen use and related knowledge.^36^ Other studies have recommended combining visual educational materials.^18,19^ A study on young people’s knowledge and awareness of skin cancer found that training programs incorporating video, brochures, and PowerPoint presentations improved behaviors related to skin self-examination.^23^ Based on these findings, video training can be considered a valuable component of educational interventions aimed at strengthening individuals’ belief in their ability to adopt skin cancer prevention practices.

This study had several limitations. First, as participation was voluntary, not all students in the targeted classes took part in the study. Second, the sample consisted of students from a single university, which limits the generalizability of the findings to the broader population of university students. Third, to ensure accurate comparison across follow-up periods, only data from participants who completed all follow-ups were included in the analysis. Fourth, the results were based on self-reported data. Nonetheless, the authors believe that the data collection tool was effective in evaluating the impact of the planned visual education program based on the HBM.

## CONCLUSION

In conclusion, the findings indicate that planned visual education based on HBM had a positive effect on perceived benefit, perceived severity, perceived susceptibility, perceived barriers, and self-efficacy. Based on these findings, it is recommended that health professionals implement skin cancer education and screening programs for young people, incorporating visual education tools grounded in the HBM—particularly those that emphasize video-based content—to promote preventive behaviors related to skin cancer.
